# Co-design of patient information leaflets for germline predisposition to cancer: recommendations for clinical practice from the UK Cancer Genetics Group (UKCGG), Cancer Research UK (CRUK) funded CanGene-CanVar Programme and the Association of Genetic Nurse Counsellors (AGNC)

**DOI:** 10.1136/jmg-2023-109440

**Published:** 2023-11-30

**Authors:** Kelly Kohut, Beverley Speight, Julie Young, Rosalind Way, Jennifer Wiggins, Laura Monje-Garcia, Diana M Eccles, Claire Foster, Lesley Turner, Katie Snape, Helen Hanson, on behalf of the CanGene-CanVar Patient Reference Panel, Caroline Dale, on behalf of the Consensus Meeting Participants, Lily Barnett

**Affiliations:** 1 Centre for Psychosocial Research in Cancer: CentRIC, University of Southampton, Southampton, UK; 2 Clinical Genetics, St George's University Hospitals NHS Foundation Trust, London, UK; 3 Clinical Genetics, Cambridge University Hospitals NHS Foundation Trust, Cambridge, UK; 4 Patient Contributor, Southampton, UK; 5 Division of Genetics and Epidemiology, Institute of Cancer Research, London, UK; 6 Cancer Genetics, The Royal Marsden NHS Foundation Trust, London, UK; 7 The St Mark's Centre for Familial Intestinal Cancer, London North West University Healthcare NHS Trust, London, UK; 8 Imperial College London, London, UK; 9 Faculty of Medicine, University of Southampton, Southampton, UK; 10 St George's University of London, London, UK

**Keywords:** Patient Care, Genetics, Genomics, Clinical Decision-Making, Information Science

## Abstract

**Background:**

Testing for germline pathogenic variants (GPVs) in cancer predisposition genes is increasingly offered as part of routine care for patients with cancer. This is often urgent in oncology clinics due to potential implications on treatment and surgical decisions. This also allows identification of family members who should be offered predictive genetic testing. In the UK, it is common practice for healthcare professionals to provide a patient information leaflet (PIL) at point of care for diagnostic genetic testing in patients with cancer, after results disclosure when a GPV is identified, and for predictive testing of at-risk relatives. Services usually create their own PIL, resulting in duplication of effort and wide variability regarding format, content, signposting and patient input in co-design and evaluation.

**Methods:**

Representatives from UK Cancer Genetics Group (UKCGG), Cancer Research UK (CRUK) funded CanGene-CanVar programme and Association of Genetic Nurse Counsellors (AGNC) held a 2-day meeting with the aim of making recommendations for clinical practice regarding co-design of PIL for germline cancer susceptibility genetic testing. Lynch syndrome and haematological malignancies were chosen as exemplar conditions.

**Results:**

Meeting participants included patient representatives including as co-chair, multidisciplinary clinicians and other experts from across the UK. High-level consensus for UK recommendations for clinical practice was reached on several aspects of PIL using digital polling, including that PIL should be offered, accessible, co-designed and evaluated with patients.

**Conclusions:**

Recommendations from the meeting are likely to be applicable for PIL co-design for a wide range of germline genetic testing scenarios.

**WHAT IS ALREADY KNOWN ON THIS TOPIC**

- Co-design is the process of involving patients, clinicians and other expert stakeholders in the process of design. Co-design is recommended for clinical pathways, guidelines and resources to include patients with lived experience as equal partners to improve services. There has been little attention and resource dedicated to co-design of patient information leaflets (PILs) for germline genetic predisposition to cancer, with wide variability in the availability and quality of PIL offered to patients across the UK.

**WHAT THIS STUDY ADDS**

- This is the first UK meeting dedicated to recommendations for clinical practice for co-design of PIL for cancer genetics.

**HOW THIS STUDY MIGHT AFFECT RESEARCH, PRACTICE OR POLICY**

- Services providing genetic testing and follow-up care for patients across the UK have agreed to use nationally developed and updated PIL to provide equity of care and improve patient experience and understanding.

## Introduction

 Implementation of the National Genomic Test Directory in England,^1^ along with growing awareness of the relevance of genomics to cancer treatment, surveillance and risk reduction,^2^,^4^ has increased the number of people with potential or confirmed germline pathogenic variants (GPV) in cancer predisposition genes. National testing and clinical management guidelines promote access and equity of care for patients. The UK Cancer Genetics Group (UKCGG) is a special interest group of The British Society for Genetic Medicine (BSGM) with multidisciplinary membership including approximately 350 clinicians and scientists. UKCGG in partnership with other stakeholders have established consensus guidelines on clinical and laboratory pathways for several indications^5^,^9^ (see UKCGG Consensus Meetings - Cancer Genetics Group). Guidelines are hosted on the UKCGG website and updated when evidence or advice changes. Some patient resources on topics such as chemoprevention and *PALB2* GPV are included. However, many of the GPV guidelines do not have associated patient resources, and there is no standard format or template for written information offered to patients.

### Current practice

There is a network of regional UK genetics services, covering large geographical areas with populations between one and five million. Geneticists and Genetic Counsellors provide education, training and expert advice to non-genetics medical and nursing colleagues to deliver ‘mainstreaming’ of germline genetic testing to eligible patients across primary and secondary care.^10^,^13^

**Figure 1:**
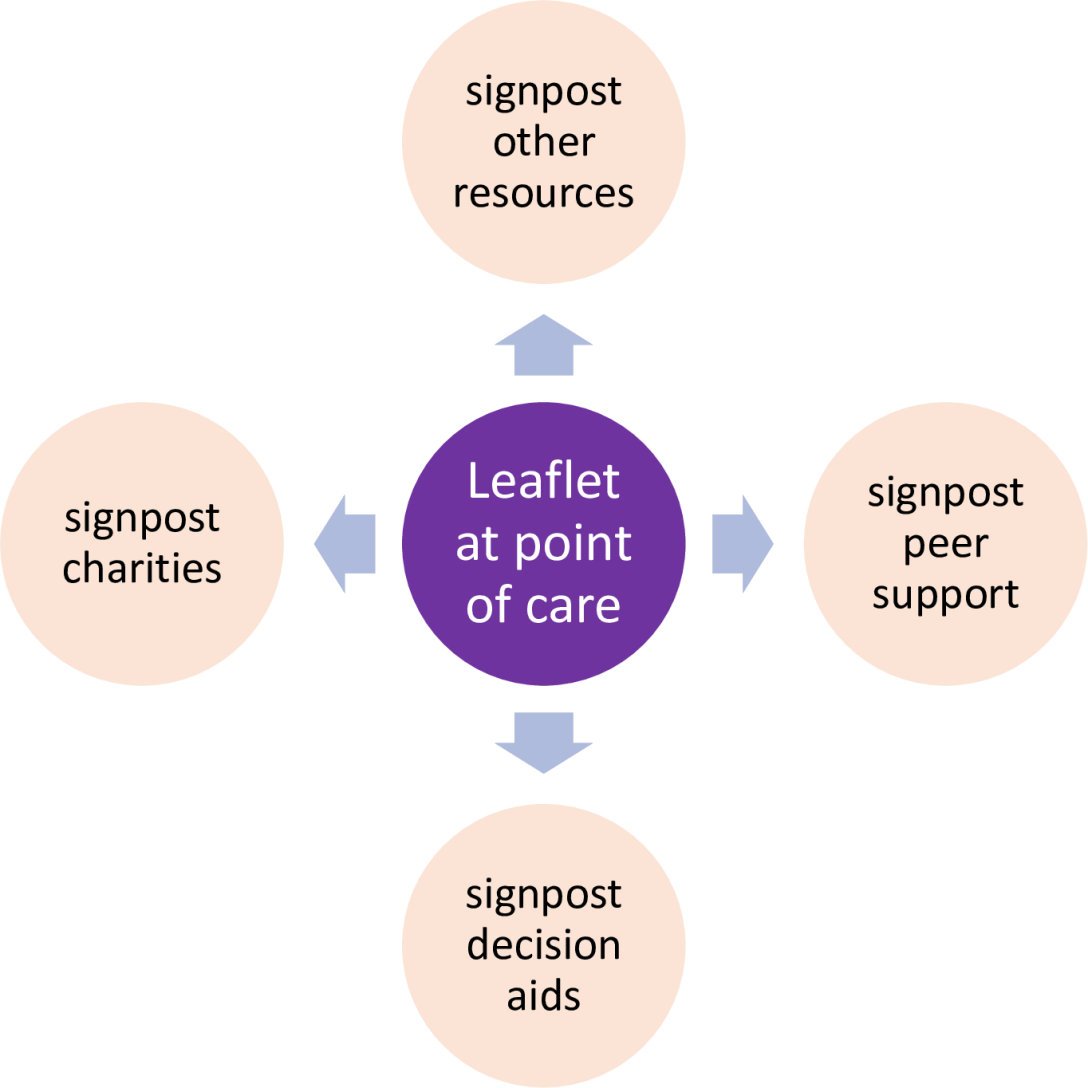
Common practice in the UK is for healthcare professionals to provide a patient information leaflet at point of care for diagnostic germline testing, after results disclosure when a germline pathogenic variant is identified, and for predictive genetic testing.

Standard practice is to offer a short patient information leaflet (PIL) at the time of genetic testing or genetic counselling in three scenarios (figure 1):

1. Diagnostic genetic/genomic testing: for patients with cancer. This includes somatic tumour testing to inform treatment and/or germline (constitutional) testing which could have familial implications due to heritable transmission of cancer susceptibility.
2. Person with a GPV: post-genetic test results. This informs cancer treatment and management and predicts future cancer risks in the patient tested and their relatives.
3. Predictive genetic testing: in at-risk relatives, for a known familial GPV. Testing is offered initially to first-degree relatives and then cascaded to the wider family.

Scenarios 1 and 2 take place in clinical genetics services and mainstream medical settings such as cancer services and haematology (conditions of the blood and bone marrow). GPVs in a cancer susceptibility gene confer increased risks of certain cancers that change over time and are influenced by factors such as gender, prior surgery and treatment, chemoprevention, risk-reducing surgery and modifiable lifestyle factors.^14^,^17^ Following genetic test results, patients usually receive appointments across primary and secondary care, at the relevant ages. Scenario 3 is the remit of specialist genetics services. For adult-onset genetic cancer susceptibility, predictive testing is typically delayed until adulthood to preserve decision-making autonomy.^18^ However, GPV in some genes such as *TP53* confer cancer risks from infancy and, therefore, testing may be performed via preimplantation genetic testing, prenatally or in childhood.

### PIL challenges and opportunities

PILs are typically developed in-house by services or taken from the public domain such as charity websites and only printed in black and white and if short enough, due to limitations in printing and administrative resources. PILs are usually paper documents distributed during clinic appointments or enclosed with patient letters copied to the general practitioner and other relevant healthcare professionals. PIL and/or letters may also include signposting with links to resources such as websites or PDF leaflets online. However, providers do not typically seek feedback about patients preferred modality or whether they have read paper PIL or accessed websites.

The genes being tested and number of eligible patients have been steadily increasing since the rollout of the NHS Genomic Medicine Service in England,^19^ with similar trends in Northern Ireland, Scotland and Wales. Demand for testing has outstripped the clinical genetics workforce which has not seen a concordant increase in capacity. Workforce planning is therefore underway,^20 21^ but genetics, oncology and haematology clinicians face extreme pressures in clinic, and waiting lists can be long. This leaves little time for robust development of PIL. Importantly, keeping PIL up to date adds extra pressure in a discipline incorporating fast-changing technology and research with evolving knowledge and guidelines. For example, the number of genes on the breast cancer panel test has increased from three to seven. Accurate risk penetrance estimates also necessitate regular review of evidence-based clinical management guidelines.

In addition to time and capacity pressures, there is a lack of standard guidance, frameworks or templates for PIL development in clinical genetics. In contrast, PILs have been legally required to accompany all medicines in the UK since 1999,^22^ with best practice guidelines including requirement to consult with target groups of patients (‘users’) to promote accessible information that is easy to understand.^23 24^ Variability in training, knowledge and skills for PIL design and user testing has led to inconsistency in the content and format of PIL, with virtually every genetics service provider using their own or none. Although there is a lack of genetics-specific guidance, other frameworks are broadly useful to inform best practice^25^,^29^ and various training resources and toolkits (table 1).

**Table 1:** List of selected guidelines, frameworks, training resources and toolkits relevant to PIL co-design

Author/publisher	Title	URL
Medicines and Healthcare Products Regulatory Agency (MHRA)	Best practice guidance on patient information leaflets (PILs)	https://assets.publishing.service.gov.uk
NHS Digital	Creating better content for users with low literacy	https://digital.nhs.uk/blog/transformation-blog/2019/creating-better-content-for-users-with-low-health-literacy
NHS England	Design principles: NHS digital service manual	https://service-manual.nhs.uk/design-system/design-principles
NHS England	Accessible Information Standard	https://www.england.nhs.uk/about/equality/equality-hub/patient-equalities-programme/equality-frameworks-and-information-standards/accessibleinfo/
Patient Information Forum (PIF) Tick	Trusted information toolkit for healthcare professionals	https://piftick.org.uk/healthcare-professionals-information/
Health Education England	Health literacy ‘how to’ guide	https://www.hee.nhs.uk/our-work/population-health/training-educational-resources
Health Education England, Health Dialogues, NHS England Department of Health, Lancashire Care NHS Foundation Trust	Making Every Contact Count (MECC)	https://www.e-lfh.org.uk/programmes/making-every-contact-count
NHS England Department of Health and Social Care	B1762: Guidance on working in partnership with people and communities	https://www.england.nhs.uk/publication/working-in-partnership-with-people-and-communities-statutory-guidance/
Alexandra Freeman. *Drug and Therapeutics Bulletin* 2019; 57:119–124	How to communicate evidence to patients	http://dx.doi.org/10.1136/dtb.2019.000008
Academy of Medical Royal Colleges	Please, write to me: Writing outpatient clinic letters to patients Guidance	https://www.aomrc.org.uk/reports-guidance/please-write-to-me-writing-outpatient-clinic-letters-to-patients-guidance/

### Co-design with patients and other experts

Patients with lived experience of genetic testing or a genetic condition are experts in their own care. They should be asked to contribute from the conception stages of research and clinical pathways and will make a thoughtful and valued impact to co-design. Although they may develop into ‘experts’ with experience on patient panels and committees, they continue to represent the wider community and advocate for increased equity, diversion and inclusion of views.^30^

### Aims

A 2-day meeting was arranged with the following aims:

1. Agree UK recommendations for clinical practice for the PIL regarding genetic cancer susceptibility testing and management in terms of content and format.
2. Take a co-design approach with patients and other experts to agree recommendations for PIL that can be adopted for specific conditions, starting with Lynch syndrome and germline genetic susceptibility to haematologic cancer, followed by GPV in other cancer susceptibility genes, and GPV in non-cancer related genes (common and rare genetic conditions).
3. Provide consistency across the UK of high-quality information given to patients accessing genetic testing and follow-up care for a GPV in a cancer susceptibility gene.
4. Minimise duplication of effort with every specialist clinical genetics or mainstream service creating their own PIL with limited time and resources to keep these updated. Accomplish this through formation of a national collaboration and working groups.
5. Create a list of trusted, up-to-date patient resources for signposting, stored centrally online via a trusted provider (eg, UKCGG) with links on other relevant websites such as GeNotes, the Genomics Education Programme and various professional resources, patient groups and charities.

## Methods

### Pre-meeting planning

The lead author (KK) submitted a proposal to seek UK consensus on recommendations for clinical practice for co-design of PIL for cancer susceptibility genetic testing and management. This was ratified at the UKCGG Executive Council Meeting on 11/10/2022. Online meetings were scheduled across two mornings. An organising committee was assembled, with all members invited to be co-chairs and named authors. The committee included representatives from clinical genetics (KK/HH/BS/KS/JW), specialist mainstream services providing genetic testing (LM-G), UKCGG Council (HH/BS/KS), a patient representative from the CRUK-funded CanGene-CanVar programme (JY) and administrative/management support from the Institute of Cancer Research (RW). The Association of Genetic Nurse Counsellors (AGNC) Chair was also engaged, agreed to co-badge the meetings and delegated a committee member to participate.

Lynch syndrome was used as an exemplar condition for the first meeting and germline predisposition to haematologic malignancies for the second. These were chosen to provide specific content, for example, of PIL content and format. Increased testing for Lynch is a current focus of NHS England, with a National Transformation Project.^31^,^33^ Germline predisposition to haematological cancer was considered during a recent UKCGG meeting resulting in publication of consensus best practice guidelines.^6^ Selection of these conditions also allowed for purposive sampling of relevant stakeholders to invite, including patients with lived experience, charities, peer support organisations, medical and academic specialists. KK invited the UK Lead Genetic Counsellor Group and the Lead Cancer Consultant Geneticist Group. All regional and specialist genetics services across the UK were asked to delegate at least one clinician for each meeting.

Registration using the online video conferencing platform https://zoom.us (‘zoom’) included expressions of interest to attend one or both meetings. Spaces were unlimited but allocated to promote representation from across the UK and include experts across the spectrum of clinical, research, policy and charity/patient support pathways. There were 10 funded patient representative places each day, with reimbursement in line with NIHR guidelines.

Relevant background reading materials and pre-meeting surveys (online supplemental file 1) were sent to participants. Survey questions assessed current practice regarding genetic and genomic testing in specialist clinical genetics and mainstream settings and use of PIL. Participants were asked to add PIL created by their services, or to which they signposted patients, to a shared Google drive folder.

### Meeting content

Following short presentations about background, best practice guidelines and existing patient information resources (see Agenda, online supplemental file 2), polls using the online platform https://community.slido.com (‘Slido’) presented consensus statements for voting regarding recommendations for clinical practice for PIL content and design. UK participants were eligible to vote. Other international experts were invited to participate but did not vote. Consensus was achieved with a threshold of 80% selecting ‘agree/strongly agree’, in accordance with the UKCGG Consensus Meetings Standard Operating Procedure (V.1, 02/12/2022, https://ukcgg.org). If consensus was not reached, the poll question could be revised in real time, but if not reached after a second vote, it was agreed that future work on this question would be required. Voting needed to be completed by at least 80% of UK participants before poll questions were closed.

Discussion and comments were encouraged to capture rich qualitative data to supplement quantitative poll data. The chat function in zoom was used, and participants could turn on their microphone and camera if they wished. The chat text was saved for descriptive analysis. The transcript was reviewed and analysed by the organising committee to identify important themes not captured in the short consensus statements displayed in the Slido polls.

## Results

### Pre-meeting surveys

Pre-meeting surveys to scope the origin and current use of PIL and other resources received low response rates: n=16/104 (15%) for the first meeting (Lynch) and n=23/147 (16%) for the second (haematology).

Results from the Lynch pre-meeting survey showed that 11/16 responders provided a PIL. Nine out of 11 were locally written and curated PIL and 2/9 were created with patient involvement. Fifteen out of 16 responders signposted patients to charities or support organisations, the vast majority to Lynch syndrome UK. In response to a question about what additional resources would be helpful, comments were made about gene-specific risks/management, as well as PIL for different stages of the genetic testing pathway.

Four out of 23 responders to the haematology pre-meeting survey indicated they provided a PIL, and these were locally written/curated. Nine out of 23 responders signposted patients to charities or support organisations. Named charities included MDS UK Patient Support Group, Leukaemia Care UK, Macmillan Cancer Support and Blood Cancer UK. Comments showed a demand for PIL to address somatic versus germline genetic variants, familial implications, predictive testing and gene-specific risks/management.

### Collation of PIL in current use

PIL in current use were added to a shared Google drive by 7/23 regional genetics services in the UK and three specialist genetics service or patient charities. These varied in length, content and format. There was a lack of patient co-design or at least notation of this on the PIL. Outreach to services that were non-responders will be undertaken by working groups overseen by the AGNC, in preparation for future work to develop condition specific PIL.

#### Meeting participants

Over 100 invitations were sent inviting patients and professionals to attend one or both meetings, share with their team and/or suggest relevant stakeholders. Interest in the meetings was universal, but availability to attend and complete the pre-meeting surveys was limited due to time pressures, clinics and other commitments. Three patients and 17 professionals attended both meetings, but only voted once (on day 2). All other participants attended one meeting and voted once in the polls. There were 48/61 engaged with polls in the first meeting and 43/57 in the second.

#### Digital polling and consensus statement agreement

Recommendations for clinical practice are presented in table 2. Detailed poll results are presented in online supplemental table. Questions were grouped into seven sections/subheadings to address the following topics: diagnostic genetic/genomic testing, patients with a GPV in a cancer susceptibility gene, predictive genetic testing, PIL format, PIL content, risk communication and communicating uncertainty.

**Table 2:** Recommendations for clinical practice from the UK Cancer Genetics Group (UKCGG), Cancer Research UK (CRUK) funded CanGene-CanVar programme and the Association of Genetic Nurse Counsellors (AGNC) on co-design of patient information leaflets (PILs) for germline predisposition to cancer

PIL indication/topic	Recommendations for clinical practiceIt should be best practice for PIL:	Suggestions from meeting discussion
Diagnostic genetic testing	To be offered to people with cancer or a pre-malignant condition being offered genetic/genomic testing	Need less detail pre-results
Pathogenic gene variant	To be offered to people who have a pathogenic variant in a cancer susceptibility gene	Mostly generic PIL+personalised letter
Predictive genetic testing	To be offered to people being offered predictive testing (in addition to a copy of their clinic letter)	At-risk relatives should be referred for genetic counselling
PIL format	To contain subheadings to make finding information easier	Should stand outEg, bold
	Subheadings to be presented in the form of questions	
	To include pictures to help explain key concepts	
PIL content	To mention psychological aspects/feelings related to having genetic testing	
	To include links to relevant charities	Check trusted
	To include links to relevant patient peer support groups	
	To include information about family planning/reproductive options, where relevant	Check phrasing with patients
	About genetic testing to present all the choices, including to do nothing/not have genetic testing	
	About genetic testing to mention rules about genetic testing and insurance	See ABI Code
	About genetic testing to mention what might happen after results	
	For people with a pathogenic gene variant to mention that more personalised information can be provided during an appointment with genetics or other specialists	Precise estimates might not be available
	To be checked using a readability tool such as SMOG with the aim of achieving a reading level of 9–11 years. Medical terms may be temporarily removed, then added back into the PIL, making sure they are clearly explained	Aim for national reading age
	To include simple explanations for any medical jargon or complex language	
	To include the term pathogenic gene variant to match the term on genetic test reports	Other descriptions can be included
	To be translated into the patient’s first language, if resources are available	
	To be reviewed by patients with lived experience of the condition	Cost for this
	To consider language and aim to be as inclusive as possible for all patients, including those with protected characteristics	Co-design with these patients
	To have a date issued and date due for review	Secure funding
Risk communication	To include information about the chances of getting cancer/pre-malignant conditions, where relevant	
	To present chances for people to get cancer/premalignant conditions with numbers as well as words (eg, showing % or a x/10 or x/100 people, not just saying ‘high’ or ‘low’ chance)	
	To include visual presentation of the chances of getting cancer/premalignant conditions, for example, icon arrays (repeated shapes showing people affected in a different colour), graphs, bar charts	Icon arrays preferred
	To include contact details for relevant healthcare professionals/services (eg, genetics, oncology, haematology)	
Communicating uncertainty	To explain uncertainty, including where it comes from (such as lack of scientific knowledge, not enough families to study) and how this might make people feel	Area for further research

See online supplemental table for more details about discussion and recommendations from meeting participants.

Consensus was reached on all statements when voting across both days was considered. The same statements were presented at both meetings. There were two statements where consensus was not reached on day 2 only, one regarding including links to peer support groups (agree/strongly agree: day 1=87%; day 2=79%+16% neutral/no opinion, online supplemental table, Section 5) and one regarding phrasing subheadings in the form of questions (agree/strongly agree: day 1=84%; day 2=65%+26% neutral/no opinion, online supplemental table, Section 4).

There was some minor revision of the statements agreed in real time on day 2 shown with tracked changes (online supplemental table). There was little opportunity to explain complicated concepts due to the character limit for Slido. Rewording was based on in-meeting feedback and aimed at increasing statement clarity.

### Descriptive summary of discussions

A descriptive summary is presented below, under poll topic heading.

1. Diagnostic genetic/genomic testing

Most genetic testing discussions occurred within clinical genetics services. This may not be representative of the proportion of tests undertaken within clinical genetics versus a mainstream setting but rather could reflect the fact that most meeting participants were from clinical genetics. High-level consensus was reached regarding the offer of a PIL at the time of diagnostic genetic testing. Chat analysis showed that participants did not feel this needed to be extensively detailed, especially since some genetic tests are broad and most patients do not have a GPV identified. A shorter PIL was suggested, which could be replaced by a longer, more specific and detailed PIL if a GPV was identified.

1. Patients with a GPV

Most genetic test results were delivered by specialist clinical genetics services, with a minority by oncology. Again, this may be representative of participant specialty rather than an overall practice in the UK. High-level consensus was reached regarding the offer of a gene-specific PIL at this stage in the pathway of care. Chat comments suggested it was acceptable for the PIL to be comprised of mostly generic information if it accompanied a personalised clinical letter.

1. Predictive genetic testing

Most discussions took place within specialist clinical genetics services. High-level consensus was reached regarding the offer of genetic counselling and a PIL at the time of predictive testing.

1. PIL format

Most people felt that up to two sides of A4 paper should be the maximum length. Chat comments showed that longer PIL, such as The Royal Marsden Beginner’s Guide to Lynch syndrome, could also be useful, but this is rarely printed due to length. High-level consensus was reached on the need to include sections with subheadings. There was verbal and chat discussion about whether PIL subheadings should be presented in the form of questions. Participants felt this could make the PIL appear more personal but could also reduce relevance for some patients, dependent on the topic.

1. PIL content

Many consensus statements on day 2 were revised live, based on participant feedback. Several referred to inclusion of certain information, such as reproductive risks. Discussion suggested some sections would not be relevant to many patients. Changes are shown in online supplemental table, mostly adding ‘where relevant’ to reflect that it would only be appropriate in specific situations, for example, involving a patient of reproductive age. For GPV in many cancer susceptibility genes, there is insufficient evidence to provide personalised risk estimates. It was felt that healthcare professionals should not overemphasise the possibility of this where data is scarce and there are no management guidelines. Preferences for terminology to describe results from cancer susceptibility gene testing ranked ‘mutation’ below gene alteration, gene change and pathogenic variant, which fits with a general trend away from using mutation in clinical practice due to its potential negative connotations.

1. Risk communication

Polling questions revealed the importance of showing visual presentations of the chance of getting cancer in the future rather than only describing risk in words. This can be achieved with numbers, pictures and graphics. Discussion highlighted the icon arrays in the NICE patient decision aid for Lynch syndrome: Should I take aspirin to reduce my chance of getting bowel cancer?^34^ as particularly helpful.

1. Communicating uncertainty

Consensus statements showed the importance of conveying the origin of uncertainty and ranked showing the range of known risks above other options. In situations where this is not possible, the chat suggested it would be acceptable to convey the amount of uncertainty in words, for example, ‘some uncertainty’ or ‘a lot of uncertainty’.

## Discussion

This was the first UK meeting dedicated to recommendations for clinical practice for PIL for testing and management of genetic cancer susceptibility. There was active participation and support from a multidisciplinary group of healthcare and academic professionals from across the UK together with patients, charities and peer support groups. Consensus was reached on all statements when poll results across both days were considered. Live discussion among presenters and participants resulted in some minor revisions to some statements on day 2. Overall, results indicated shared enthusiasm to collaborate and make best use of limited resources to improve the quality, usefulness and consistency of PIL offered to patients. Pre-meeting survey response rate was low, reflecting time pressure from attendees. The limited responses revealed variability in PIL use, format and content in the context of testing and management of genetic cancer susceptibility. There was limited evidence of patient co-design and many PILs contained complex terminology resulting in a high reading level, with limited use of visual presentation of cancer risks and communication about uncertainty. This was not surprising, given the stretched resources in healthcare services making co-development of robust PIL that meet the NHS Accessible Information Standard^26^ and contain up-to-date, evidence-based information a challenge, particularly for genetics which is a rapidly developing specialty with an ever-increasing relevance to various points of care for patients in virtually all areas of medicine. Variability across services and geographies has made delivery of best practice guidelines challenging^9^ and predictably patient experience with PIL has also been mixed, from not receiving PIL at all to PIL ranging from low to excellent quality and usefulness. Factors including ease of understanding, experience and emotions can also affect how meaningful PIL are for patients.^35 36^ This is often unexplored when there is only one version available and no evaluation by patients who might benefit the most from more simple PIL,^37^ although ‘easy read’ versions that rely mostly on pictures are starting to be developed as options (eg, see NHS England guide to whole genome sequencing, The Eve Appeal Lynch Syndrome Guide, Beyond Words colonoscopy PIL).

PIL can be improved and made easier to read by using validated readability checker tools such as Flesch-Kincaid (FK), Simple Measure of Gobbledygook (SMOG), Gunning fog index (GFI), Fry, FORCAST and Flesch Reading Ease (FRE),^38^ aiming for the national average reading age of 9–11 years. However, better satisfaction have been achieved by involving patients in co-design and evaluating impact.^39 40^ Gold standard PIL would be tailored to individuals due to the highly personal nature of health decisions, for example, by using computer software,^41^ although this would require significant research and resource to implement and was recognised as beyond the scope of our recommendations at the current time. National collaboration is an efficient way of pooling limited resources to co-design good-quality, useful PIL rather than have many different services either duplicating efforts to produce similar resources or not securing the time and resource to create and use PIL at all.

Key recommendations for clinical practice from patients and stakeholders contributing to polling and discussions (table 2) are summarised as:

1. Patients should be offered a PIL, alongside their personalised clinic letter, during the genetic testing process (diagnostic and predictive)
2. PIL should be as inclusive as possible, with attention to readability, separate sections and inclusion of visuals (such as using numbers as well as simple words, pictures, icon arrays)
3. PIL should include date of creation and next review and signpost to relevant charities/support organisations/healthcare services
4. Patients with lived experience of the condition should be invited to co-design and review PIL.

### Strengths and limitations

A major strength of these meetings was inclusion of patients with lived experience of cancer, haematologic conditions and/or genetic testing and representation from patient groups and charities. The virtual meeting format removed cost and time restrictions associated with in-person meetings and therefore encouraged UK-wide representation from clinical genetics services and other specialties including oncology and haematology in addition to expert stakeholders. The group was multidisciplinary which encouraged lively discussion with varied perspectives, views and recommendations based on personal experience, local infrastructure and pathways.

Partnership between UKCGG, CanGene-CanVar and AGNC along with specific cancer and genetic patient groups and charities allowed organisations with shared goals to pool resources including finance, staff and time to maximise efficiency and output.

Funding was only available for 10 patients per day; this included remuneration for time spent preparing and attending the meetings. Although not all claimed this offer of reimbursement, funding must be available at the planning stage, which therefore limited the number of patients invited. It would have been beneficial to have more patients to increase the number and diversity of viewpoints. This will be the focus of future funding requests for follow-on work co-designing condition-specific leaflets.

Only two conditions, Lynch and haematological malignancies were used to consider specific PIL content. It was challenging to fully consider the complexities of these two conditions given the various genes and corresponding guidelines. Further, more focused working groups will be convened to fully explore the views and preferences for these patient groups before moving onto other conditions, applying what has been learnt to the generic PIL template design. Additional resource is required and will be the subject of future funding applications.

## Conclusions

Regarding the aims of the meetings:

1. UK consensus was achieved on recommendations for clinical practice for PIL content and format regarding genetic cancer susceptibility testing and management.
2. A co-design approach was taken with patients and other expert stakeholders.
3. The recommendations will promote consistency across the UK of high-quality information given to patients.
4. Duplication of effort has been reduced through formation of a national collaboration and working groups.
5. Work has been initiated to create a list of trusted, up-to-date external resources stored centrally online.

This work provides a unique contribution to the literature, reporting the first UK meeting on co-design of PIL for cancer genetics. National collaboration was effective to maximise resources with the shared aim of improving patient care and resources.

### Future work

A collaboration has been initiated with the newly formed AGNC Working Group on PIL to maximise output by adapting the UKCGG PIL consensus template for other genetic conditions, starting with cancer susceptibility genes and then considering non-cancer-related genetic conditions.

Charities and patient groups relevant to the condition-specific leaflets will be invited to review the content and put the PIL through their internal processes to consider co-badging. This could increase trust from some patients who have confidence in information provided by patient-led organisations rather than government, medical or academic institutions.

PIL will be hosted on the UKCGG website, freely accessible alongside current clinical guidelines for GPV in cancer susceptibility genes. A publication date and review date will be noted in the PIL footer. Future funding will be sought to ensure dedicated time to update the PIL when needed, with input from a diverse group including patients, charities and other expert stakeholders.

## supplementary material

10.1136/jmg-2023-109440 online supplemental file 1

10.1136/jmg-2023-109440 online supplemental file 2

10.1136/jmg-2023-109440 online supplemental file 3

## Data Availability

All data relevant to the study are included in the article or uploaded as supplementary information.
