# Supplementary material for: Co-design of patient information leaflets for germline predisposition to cancer: recommendations for clinical practice from the UK Cancer Genetics Group (UKCGG), Cancer Research UK (CRUK) funded CanGene-CanVar Programme and the Association of Genetic Nurse Counsellors (AGNC)
Source: J Med Genet. 2023 Nov 30;61(2):e109440. doi: 10.1136/jmg-2023-109440 (PMC11881048; doi:10.1136/jmg-2023-109440)
Supplement: online supplemental file 1 [file jmg-61-2-s001.pdf]

Supplementary File 1. Pre-meeting surveys

Pre-meeting survey for Day 1: Lynch syndrome
Patient resources - genetic testing for Lynch Syndrome

Question Title

1. Do you provide patient information leaflets in your clinical practice relating to genetic testing for Lynch?

- Yes
- No

Question Title

2. Do these include locally written/curated patient information leaflets?

- Yes
- No
- Unsure
- I don't use patient information leaflets regarding genetic testing for Lynch (go to question 3)

2a) Has there been any patient input or involvement in developing the leaflets?
Yes
No
Unsure
Comments: [free text]

Question Title

3. Do you ever signpost patients to charities or support organisations regarding Lynch?

- Yes
- No

Question Title

4. What charities/support organisations do you signpost to?

Question Title

5. What additional patient leaflets/resources would be helpful in your clinical practice relating to genetic testing for Lynch?

6. Please add any leaflets currently used by your service to the [google drive] or send to the meeting organisers as an attachment. These will be collated and shared for information prior to the meeting.

Pre-meeting survey for Day 2: Haematology Patient resources - genetic testing in haematological malignancy

Question Title

1. Do you provide patient information leaflets in your clinical practice relating to genetic testing in haematological malignancy?

- Yes
- No

Question Title

2. Do these include locally written/curated patient information leaflets?

- Yes
- No
- Unsure
- I don't use patient information leaflets regarding genetic testing in haematological malignancy

Question Title

3. Do you ever signpost patients to charities or support organisations regarding haem/myeloid disease?

- Yes
- No

Question Title

4. What charities/support organisations do you signpost to?

Question Title

5. What additional patient leaflets/resources would be helpful in your clinical practice relating to genetic testing in the haematological setting?

Done
