# Supplementary material for: Co-design of patient information leaflets for germline predisposition to cancer: recommendations for clinical practice from the UK Cancer Genetics Group (UKCGG), Cancer Research UK (CRUK) funded CanGene-CanVar Programme and the Association of Genetic Nurse Counsellors (AGNC)
Source: J Med Genet. 2023 Nov 30;61(2):e109440. doi: 10.1136/jmg-2023-109440 (PMC11881048; doi:10.1136/jmg-2023-109440)
Supplement: online supplemental file 2 [file jmg-61-2-s002.pdf]

UK Cancer Genetics Group National Consensus Meeting:

Co-design of patient leaflets

Online consensus meeting to be held over two mornings:

Thursday 16/03/2023, 09:30-12:30

Friday 17/03/2023, 09:30-12:30

Meeting registration

Registration on the zoom platform is required prior to joining the meeting. Please register by clicking the relevant link below:

- To register for **16/03/2023:** https://the-icr.zoom.us/meeting/register/u5Iqc-6hrj0sGdGch52dlz5KmA6GS5hCHQbm
- To register for **17/03/2023**: https://the-icr.zoom.us/meeting/register/u5MrcOCtrzkvHtRLwouReO9kmn4J7DVfnghC

Registration is now open for this meeting. Spaces will be limited to 500 and allocated to promote representation from all clinical genetics services for England, Scotland, Wales and Northern Ireland along with individuals with roles across the spectrum of the clinical, research, policy and charity/patient support pathways. One patient representative will be a co-chair and there will approximately 10 other patient places for each day, with reimbursement for their time in line with NIHR guidelines.

Those who register their interest but are unfortunately unable to be offered a place due to limited spaces and requirement for diverse representation will be able to access the relevant materials, presentations and consensus documents both prior to and following the meeting. In registering your interest for this event, please note you are confirming your intention to attend all sessions on one or both dates, in order for the consensus outcomes to represent all delegates and specialties.

Pre-meeting reading

Delegates are asked to review the following documents before the meeting:

If attending either day:

1. Hastings Ward et al. 2022 Research participants: critical friends, agents for change https://rdcu.be/c6ZPe
2. Winton Centre for Risk and Evidence Communication/University of Cambridge leaflet about genetic results report template project
3. *NHS Statutory Guidance B1762, Working in Partnership with People and Communities https://www.england.nhs.uk/wp-content/uploads/2022/07/B1762-guidance-on-working-in-partnership-with-people-and-communities.pdf

*This is a long document, and you do not need to read it all. Please look at the following 2 pages:

- Title page
- Page 8, drawing attention to Priority 6: Provide clear and accessible information to the public
- (Optional)- if you would like to browse the easy read version https://www.england.nhs.uk/wp-content/uploads/2022/07/Easy-read-Working-with-People-and-Communities.pdf

4. NHS Design Principles

If attending the Lynch day on 16/3:

1. UKCGG Lynch Specific Patient Guidelines (please note these are currently being updated and it is always best to go to the website to access the most current versions) UKCGG leaflets and guidelines - Cancer Genetics Group

If attending the Haem day on 17/3:

1. Speight et al. 2022 Germline predisposition to haematological malignancies: Best practice consensus guidelines from the UK Cancer Genetics Group (UKCGG), CanGene-CanVar and the NHS England Haematological Oncology Working Group - PubMed (nih.gov)
2. Cambridge University Hospital patient information leaflets

Collaborators

This meeting is supported with funding from:

- UK Cancer Genetics Group (UKCGG)
- CanGene-CanVar (CGCV) Programme funded by Cancer Research UK

The resultant leaflets will be co-badged with logos from the following, subject to review and approval:

- The Association of Genetic Nurses and Counsellors (AGNC)
- NHS England Genomics Unit

Patient groups/charities, including condition-specific (to be confirmed, all stakeholders to suggest any additional delegates, please):

- Gene People

Lynch Syndrome:

- Lynch Syndrome UK
- Bowel Cancer UK
- Peaches Womb Cancer Trust
- The Eve Appeal
- Macmillan Cancer Support

Haematological conditions:

- Leukaemia Care
- MDS-UK Patient Support Group

The leaflets will be hosted on the UKCGG website, freely accessible to all alongside the current clinical guidelines. A publication date and review date will be noted on leaflets. Future funding will

be sought to ensure dedicated time to update the leaflets when needed, with input from patients and other expert stakeholders.

Background

The implementation of the National Genomic Test Directory in England along with increasing public and professional awareness of the relevance of genomics to cancer screening, prevention, early detection and treatment has led to an increasing number of people with either potential or confirmed germline predisposition to malignant and pre-malignant conditions. National UK consensus on clinical management pathways is required for both the affected individual and their relatives. The UKCGG has established consensus guidelines on clinical and laboratory pathways for several indications (see UKCGG Consensus Meetings - Cancer Genetics Group).

A short patient information leaflet is standardly given or enclosed with a letter at the time of genetic testing or genetic counselling in three scenarios:

1. **Diagnostic genetic/genomic testing:** for a person diagnosed with cancer or a pre-malignant condition
2. **Person with a pathogenic gene variant:** follow-up appointment and onward referrals after genetic testing has found a pathogenic gene variant
3. **Predictive genetic testing:** for an at-risk relative of a person who carries a known pathogenic gene variant

Scenarios 1 & 2 take place when genetic/genomic testing is offered in clinical genetics services as well as mainstream medical settings including oncology and haematology. People with a pathogenic gene variant have a lifelong condition and will usually receive referrals and care in the community across primary and secondary care over time, at the relevant ages.
Scenario 3 is the remit of clinical genetics service.

Aims:

1. Achieve UK consensus on the minimum data set regarding what information should be included in patient leaflets regarding genetic/genomic testing for cancer susceptibility or a pre-malignant condition and how this information should be displayed. Take a co-design approach with patients and other expert stakeholders to create a patient information leaflet template that can be adapted and populated with condition-specific information, starting with Lynch syndrome and haematological conditions followed by other cancer susceptibility syndromes.
2. Provide consistency of information given to patients accessing genetic testing and follow-up care across the UK
3. Minimise duplication of effort with every clinical genetics or mainstream service creating their own leaflets with limited time and resource to keep these updated
4. Create a list of trusted, up-to-date resources that can be signposted for patients

Future steps: collaborate with the AGNC working group on patient leaflets to maximise output, by sharing the patient information leaflet template to adapt for other, non-cancer genetic conditions.

Methods

Online workshops held across two mornings, using Lynch Syndrome as an exemplar condition on Day 1 and Haematological conditions as the exemplar for Day 2. Digital polls using Slido will present consensus statements. Delegates from the UK will vote on these polls until consensus is achieved

(threshold 80%, in accordance with the UKCGG Consensus Meetings SOP v1 02/12/2022). If consensus is not reached, the poll question wording may be modified in real time, but if consensus is not reached after a second vote, further work on this question may be required in a separate, future meeting. Voting should be undertaken by at least 80% of UK attendees. Other international experts may give presentations and attend the meeting but will not vote in the polls for UK consensus.

Post-meeting publication, materials and papers:

- Meeting materials, agendas, outcomes and presentations (subject to speaker consent) should be made available through the UKCGG website both prior and subsequent to the meeting
- UKCGG social media and comms representative can undertake publicity work relating to the meeting via UKCGG website and social media channels
- UKCGG and CGCV (CRUK) support should be acknowledged in all subsequent publications pertaining to this work
- Co-chairs and members of the core organising committee will be invited to be named authors on manuscript(s) pertaining to this work. All voting delegates will be listed in a consortium authorship.

Agenda:

DAY 1

Thursday 16/03/2023: Lynch Syndrome focus

**09:30-09:50**	**Kelly Kohut,** *Co-Chair, Lead Consultant Genetic Counsellor, St George's University Hospitals NHS Trust & PhD student, CanGene-CanVar, University of Southamptom*	Background & rationale for meeting<br>Ground rules<br>Pre-meeting survey results<br>Guidelines & frameworks for information design for patient leaflets
**09:50-10:00**	**Dr Helen Hanson,** *Chair of UKCGG Council & Joint Lead Consultant for Cancer Genetics, St George's University Hospitals NHS Trust*	Remit of UKCGG<br>Consensus Meetings SOP<br>Clinical guidelines, patient resources & other information on UKCGG website
**10:00-10:15**	**Julie Young, Patient Co-Chair,** *CanGene-CanVar Patient Reference Panel*	Lived experience of receiving patient leaflets and signposting to patient resources:<br>What was helpful? Not so helpful? Was there anything missing to support you?

Presentations about existing resources		
**10:15-10:20**	**Jennifer Wiggins,** *Co-Chair, Senior Genetic Counsellor, The Royal Marsden NHS Foundation Trust*	The Royal Marsden Beginner's Guide to Lynch Syndrome
**10:20-10:25**	**Laura Monje-Garcia,** *National Lead Nurse for Lynch Transformation Project & St Mark's Hospital*<br><br>**Tracy Smith,** *Lynch Syndrome UK*	The Lynch Patient Passport
**10:25-10:30**	***Lydia Brain,*** *Communications and Media Manager, The Eve Appeal*<br>**Tracey Miles**, *Associate Director of Nursing and Midwifery, South West GMSA & Ask Eve Cancer Information Nurse*	A Guide to Lynch Syndrome
**10:30-10:35**	Dr. Stan Shepherd, CEO Instant Access Medical Limited	Lynch patient dashboard app
**10:35-10:40**	**Helen White,** *Patient Representative*	The Peaches Womb Cancer Trust
10:45-11:00	**Break**	
**11:00-12:15**	Focused discussions/in meeting digital consensus polls using Slido (*Note: the polls will be the same tomorrow – if you are attending both days please vote on Day 2. You only need to vote once. Only UK delegates will vote in polls, but output will be shared with colleagues from Ireland and other countries*)	
**12:15-12:30**	**Co-Chairs: Kelly Kohut, Dr Helen Hanson Jennifer Wiggins Bev Speight Patient Co-Chair**	Round-up<br>Plans for next steps & dissemination of outputs
**12:30**	Meeting finishes	

DAY 2

Friday 17/03/2023: Haematological conditions focus

**09:30-09:40**	**Bev Speight,** *Co-Chair, Treasurer, UKCGG Council & Principal Genetic Counsellor, Cambridge University Hospitals NHS Foundation Trust*	Background & rationale for meeting<br>Ground rules<br>Pre-meeting survey results
**09:40-09:50**	**Dr Katie Snape,** *Secretary of UKCGG Council & Joint Lead Consultant for Cancer Genetics, St George's University Hospitals NHS Trust*	Remit of UKCGG<br>Consensus Meetings SOP<br>Clinical guidelines, patient resources & other information on UKCGG website
**09:50-10:00**	**Julie Young, Patient Co-Chair,** *CanGene-CanVar Patient Reference Panel*	Lived experience of receiving patient leaflets and signposting to patient resources:<br>What was helpful? Not so helpful? Was there anything missing to support you?
**Presentations about existing resources**		
**10:00-10:05**	**Bev Speight,** *Co-Chair, Treasurer, UKCGG Council & Principal Genetic Counsellor, Cambridge University Hospitals NHS Foundation Trust*	Patient leaflets
**10:05-10:10**	**Charlotte Martin,** *Leukaemia Care*	Unmet needs in the patient information space
**10:10-10:25**	**Tilly Tilbrook,** *MDS-UK*	Leaflets in current use
**10.25-10.30**	**Celine Lewis,** *Senior Research Fellow in Genomics, NIHR Advanced Fellow, Population, Policy &*	Designing animations for genomics<br>http://www.ucl.ac.uk/child-health<br>*UCL profile here |Google scholar here |Twitter profile here*

	Practice Department, University College London Great Ormond Street Institute of Child Health	*Links to 'My Genome Sequence' animations part 1 and part 2*
10:30-10:45	**Break**	
**10:45-12:15**	Focused discussions/in meeting digital consensus polls using Slido<br>*(Note: the polls will be the same as yesterday – if you are attending both days please vote on Day 2. You only need to vote once. Only UK delegates will vote in polls, but output will be shared with colleagues from Ireland and other countries)*	
**12:15-12:30**	**Co-Chairs: Kelly Kohut, Dr Katie Snape<br>Bev Speight<br>Patient Co-Chair**	Round-up<br>Plans for next steps & dissemination of outputs
**12:30**	Meeting finishes	
