# Supplementary material for: Co-design of patient information leaflets for germline predisposition to cancer: recommendations for clinical practice from the UK Cancer Genetics Group (UKCGG), Cancer Research UK (CRUK) funded CanGene-CanVar Programme and the Association of Genetic Nurse Counsellors (AGNC)
Source: J Med Genet. 2023 Nov 30;61(2):e109440. doi: 10.1136/jmg-2023-109440 (PMC11881048; doi:10.1136/jmg-2023-109440)
Supplement: online supplemental file 3 [file jmg-61-2-s003.pdf]

Supplementary Table. Questions presented to participants using the digital polling platform https://community.slido.com **('Slido').** Instructions for consensus statement questions were 'Please state your level of agreement with the following statement'. Choices were strongly disagree, disagree, neutral/no opinion, agree, strongly agree, I don't know. Agree and strongly agree were added together to confirm if the threshold of 80% agreement for consensus was reached. Instructions for other voting or rating questions are noted in the tables.
Rewording of some questions was performed in real time on Day 2, based on feedback from the digital chat and verbal discussions. This is indicated by ~~strikethrough~~ of the original wording and new wording presented in **bold**.

1. Diagnostic genetic/genomic testing:

For patients: Who discussed genetic testing with you?
Or: who discusses genetic testing with patients at your centre?

Instructions: Please select all that apply	**Day 1 (Lynch)** Number of votes (%)	**Day 2 (Haematology)** Number of votes (%)
Genetic Counsellor	29 (76%)	21 (58%)
Consultant Geneticist	18 (47%)	22 (61%)
Clinical Nurse Specialist	11 (29%)	13 (36%)
Nurse	3 (8%)	4 (11%)
Registrar/Junior Doctor	11 (29%)	14 (39%)
Consultant Oncologist	11 (29%)	12 (33%)
Consultant Surgeon	9 (24%)	5 (14%)
Consultant Haematologist	4 (11%)	15 (42%)
Not applicable	3 (8%)	0
Other	0	0
Other	3 (8%)	1 (3%)

It should be best practice for people with cancer or a pre-malignant condition being offered genetic/genomic testing to be ~~given~~ **offered** a patient information leaflet.
(Agree/Strongly agree: Day 1= 87%; Day 2= 89%)

2. People with a pathogenic gene variant (mutation) identified:

For Patients: Who told you the results of genetic testing?
Or: who tells patients results at your centre?

Instructions: Please select all that apply	**Day 1 (Lynch)** Number of votes (%)	**Day 2 (Haematology)** Number of votes (%)
Genetic Counsellor	32 (82%)	25 (68%)
Consultant Geneticist	21 (54%)	26 (70%)
Clinical Nurse Specialist	11 (28%)	12 (32%)

Nurse	2 (5%)	2 (8%)
Registrar/Junior Doctor	13 (33%)	17 (46%)
Consultant Oncologist	11 (28%)	14 (38%)
Consultant Surgeon	9 (23%)	5 (14%)
Consultant Haematologist	5 (13%)	15 (41%)
Not applicable	5 (13%)	4 (11%)
I don't know	0	2 (5%)
Other	4 (10%)	0

It should be best practice for people who have had a pathogenic variant in a cancer susceptibility gene identified to be ~~given~~ **offered** a gene-specific patient information leaflet.
(Agree/Strongly agree: Day 1= 86%; Day 2=96%)

3. Predictive genetic testing

For patients: Who discussed predictive genetic testing with you?
Or: who discusses predictive genetic testing at your centre?

Instructions:<br>*Please select all that apply*	**Day 1 (Lynch)**<br>Number of votes (%)	**Day 2 (Haematology)**<br>Number of votes (%)
Genetic Counsellor	31 (78%)	24 (65%)
Consultant Geneticist	19 (48%)	23 (62%)
Clinical Nurse Specialist	4 (10%)	2 (5%)
Nurse	0	0
Registrar/Junior Doctor	9 (23%)	9 (24%)
Consultant Oncologist	2 (5%)	0
Consultant Surgeon	1 (3%)	0
Consultant Haematologist	1 (3%)	5 (14%)
Not applicable	5 (13%)	2 (8%)
I don't know	1 (3%)	3 (8%)
Other	2 (5%)	0

It should be best practice for at-risk relatives who do not have a known diagnosis to be referred for genetic counselling prior to predictive genetic testing.
(Agree/Strongly agree: Day 1= 90%; Day 2=88%)

It should be best practice for people being offered predictive genetic testing to be ~~given~~ **offered** a gene-specific patient information leaflet (in addition to a copy of their clinic letter).
(Agree/Strongly agree: Day 1= 91%; Day 2=87%)

4. Leaflet format

In terms of number of pages, what do you think should be the maximum length for a patient information leaflet about genetic testing or genetic testing results?

Instructions: Please select one option	**Day 1 (Lynch)** Number of votes (%)	**Day 2 (Haematology)** Number of votes (%)
One side of one piece of paper (A4 size)	5 (12%)	5 (13%)
Both sides of one piece of paper	15 (37%)	20 (50%)
3 to 4 pages long	13 (32%)	5 (13%)
Longer than 4 pages	2 (5%)	1 (3%)
Neutral/no opinion	6 (15%)	8 (20%)
I don't know	0	1 (3%)

It should be best practice for patient information leaflets to contain subheadings. These should stand out (for example, using bold text) to make finding information easier.
(Agree/Strongly agree: Day 1= 100%; Day 2=95%)

It should be best practice for patient information leaflet subheadings to be presented in the form of questions. For example, 'Why am I being offering a germline genetic test?' instead of, 'Germline genetic testing'.
(Agree/Strongly agree: Day 1= 84%; Day 2=65% + 26% neutral/no opinion)

It should be best practice for patient information leaflets to include pictures to help explain key concepts.
(Agree/Strongly agree: Day 1= 93%; Day 2=84%)

5. **Leaflet content**

It should be best practice for patient information leaflets to mention the psychological aspects/feelings that people might have when they have genetic testing or receive results.
(Agree/Strongly agree: Day 1= 85%; Day 2=90%)

It should be best practice for patient information leaflets to include links to relevant charities.
(Agree/Strongly agree: Day 1= 95%; Day 2=95%)

It should be best practice for patient information leaflets to include links to relevant patient peer support groups.
(Agree/Strongly agree: Day 1= 87%; Day 2=79% + 16% neutral/no opinion)

It should be best practice for patient information leaflets to include information about family planning/reproductive options**, where relevant.**
(Agree/Strongly agree: Day 1= 87%; Day 2=92%)

It should be best practice for patient information leaflets to mention diet and lifestyle factors that might give people a higher or lower chance of getting cancer or a pre-malignant condition in the future**, where relevant**.
(Agree/Strongly agree: Day 1= 90%; Day 2=84%)

It should be best practice for patient information leaflets about diagnostic or predictive genetic testing to present all the choices available, including the choice to do nothing/not have genetic testing.

(Agree/Strongly agree: Day 1= 97%; Day 2=97%)

It should be best practice for patient information leaflets about diagnostic or predictive genetic testing to mention rules about genetic testing and insurance.
(Agree/Strongly agree: Day 1= 84%; Day 2=90%)

It should be best practice for patient information leaflets about diagnostic or predictive genetic testing to mention what might happen after results.
(Agree/Strongly agree: Day 1= 93%; Day 2=98%)

It should be best practice for patient information leaflets for people who have a pathogenic gene variant identified to mention that more personalised information can be provided during an appointment with genetics or other specialists.
(Agree/Strongly agree: Day 1= 90%; Day 2=95%)

It should be best practice for patient information leaflets to be checked using a readability tool such as SMOG with the aim of achieving a reading level of 9-11 years. Medical terms ~~explained in the leaflet may need to be removed to achieve this~~ **may be temporarily removed, then added back into the leaflet, making sure they are clearly explained.**
(Agree/Strongly agree: Day 1= 86%; Day 2=95%)

It should be best practice for patient information leaflets to include simple explanations for any medical jargon or complex language.
(Agree/Strongly agree: Day 1= 98%; Day 2=94%)

Pathogenic gene variant is the term used on genetic test reports. This has also been called a mutation, gene alteration or gene change.

Instructions: Please rank your preferred order of preference for the term that should be used on patient information leaflets.	**Day 1 (Lynch)**	**Day 2 (Haematology)**
Gene alteration	4.9	4.9
Gene change	4.7	4.2
Pathogenic gene variant	3.8	3.9
Mutation	2.6	3.1
Neutral/no opinion	0.5	0.2
I don't know	0.4	0.1

It should be best practice for patient information leaflets to be translated into the patient's first language, if resources are available.
(Agree/Strongly agree: Day 1= 87%; Day 2=95%)

It should be best practice for patient information leaflets to be reviewed by patients with lived experience of the condition.
(Agree/Strongly agree: Day 1= 91%; Day 2=95%)

It should be best practice for patient information leaflets to consider the language used and aim to be as inclusive as possible for all patients, including those with protected characteristics.
(Agree/Strongly agree: Day 1= 95%; Day 2=95%)

It should be best practice for patient information leaflets to have a date issued and date due for review.
(Agree/Strongly agree: Day 1= 100%; 95%)

6. Risk communication:

It should be best practice for patient information leaflets to include information about the chances of getting cancer/pre-malignant conditions**, where relevant**.
(Agree/Strongly agree: Day 1= 92%; Day 2=95%)

It should be best practice for patient information leaflets to present chances for people to get cancer/premalignant conditions with numbers as well as words (for example, showing % or a x/10 or x/100 people, not just saying 'high' or 'low' chance).
(Agree/Strongly agree: Day 1= 86%; Day 2=86%)

It should be best practice for patient information leaflets to include visual presentation of the chances of getting cancer/premalignant conditions, for example icon arrays (repeated shapes showing people affected in a different colour), graphs, bar charts.
(Agree/Strongly agree: Day 1= 89%; Day 2=84%)

It should be best practice for patient information leaflets to include contact details for relevant health care professionals/services (for example, genetics, oncology, haematology).
(Agree/Strongly agree: Day 1= 86%; Day 2=88%)

7. Communicating uncertainty:

It should be best practice for uncertainty to be explained, including where it comes from (such as lack of scientific knowledge, not enough families to study) and how this might make people feel.
(Agree/Strongly agree: Day 1= 91%; Day 2=95%)

If there is uncertainty about the chances of getting cancer/premalignant conditions for people, how should this be shown in the patient information leaflet?

*Instructions**:** Please rank in order of preference.*	**Day 1 (Lynch)**	**Day 2 (Haematology)**
Range of risk	4.6	4.6
Confidence intervals	2.3	2.4
I don't know	1.1	1.1
Don't show this	1.1	0.9
Neutral/no opinion	0.9	0.6
